# Depressive Symptoms, Pain, and Quality of Life among Patients with Nonalcohol-Related Chronic Pancreatitis

**DOI:** 10.1155/2012/978646

**Published:** 2012-11-27

**Authors:** Wendy E. Balliet, Shenelle Edwards-Hampton, Jeffery J. Borckardt, Katherine Morgan, David Adams, Stefanie Owczarski, Alok Madan, Sarah K. Galloway, Eva R. Serber, Robert Malcolm

**Affiliations:** ^1^Division of Biobehavioral Medicine, Department of Psychiatry and Behavioral Sciences, Medical University of South Carolina, Charleston, SC 29425, USA; ^2^Division of General and Gastroenterology Surgery, Department of Medicine, Medical University of South Carolina, Charleston, SC 29425, USA

## Abstract

*Objective*. The present study was conducted to determine if depressive symptoms were associated with variability in pain perception and quality of life among patients with nonalcohol-related chronic pancreatitis. *Methods.* The research design was cross-sectional, and self-report data was collected from 692 patients with nonalcohol-related, intractable pancreatitis. The mean age of the sample was 52.6 (*SD* = 14.7); 41% of the sample were male. Participants completed the MOS SF12 Quality of Life Measure, the Center for Epidemiological Studies 10-item Depression Scale (CESD), and a numeric rating scale measure of “pain on average” from the Brief Pain Inventory. *Results.* Depressive symptoms were significantly related to participants' reports of increased pain and decreased quality of life. The mean CESD score of the sample was 10.6 (*SD* = 6.5) and 52% of the sample scored above the clinical cutoff for the presence of significant depressive symptomology. Patients scoring above the clinical cutoff on the depression screening measure rated their pain as significantly higher than those below the cutoff (*P* < 0.0001) and had significantly lower physical quality of life (*P* < 0.0001) and lower mental quality of life (*P* < 0.0001). *Conclusion.* Although causality cannot be determined based on cross-sectional, correlational data, findings suggest that among patients with nonalcoholic pancreatitis, the presence of depressive symptoms is common and may be a risk factor associated with increased pain and decreased quality of life. Thus, routine screening for depressive symptomology among patients with nonalcoholic pancreatitis may be warranted.

## 1. Introduction 

Chronic pancreatitis (CP) is a long-term, often debilitating medical condition that drastically impacts the health and quality of life of affected patients. The disease involves persistent inflammation of the pancreas, causing the hallmark symptom of severe abdominal pain in 80–90 percent of patients [1, 2]. Initial symptoms are often described as pancreatitis “attacks” characterized by episodes of extreme acute pain. Progression of the disease is marked by increased incidence and severity of pain attacks, culminating in chronic pain, nausea, and vomiting, which significantly impairs physical and psychosocial functioning. In addition to debilitating pain symptoms, CP patients frequently report an array of distressing gastrointestinal symptoms, including malabsorption, fat intolerance, anorexia, diarrhea, jaundice, and progressive impairment of pancreatic enzyme output. Insufficient enzyme production often leads to additional complications, such as endocrine insufficiency and insulin dependence [3–5]. Untreated chronic pancreatitis is associated with high morbidity and mortality rates; long-term improvement of pain symptoms without surgical intervention is uncommon [5, 6].

Importantly, in addition to multiple distressing physical symptoms, individuals with chronic pancreatitis report significant difficulties in social and emotional functioning. The unique interplay between physical and psychosocial symptoms of CP is not well-understood. Higher rates of clinically significant depression and anxiety amongst CP patients (in which the etiology is frequently due to alcohol use) are documented in the literature, and various social and physical variables associated with CP likely interact to create distressing symptoms and reports of reduced quality of life [7–14]. For instance, chronic pain has been documented as the most important factor in causing distress in CP patients [15, 16]. Untreated social, emotional, and behavioral symptoms may also lead to disease progression and exacerbate pain and gastrointestinal symptoms in CP patients. 

Further, the etiology of patient's CP may contribute to reported emotional, social, and physical symptoms. Alcoholism is the most common cause for nonobstructive pancreatitis and is thought to account for 70% of cases of CP [17]. Treatment of alcohol-related pancreatitis is often challenging, due to multiple problems associated with alcohol misuse, including dependency, psychosocial difficulties, mood symptoms, and physical complications of malnutrition and hepatic insufficiency [18, 19]. More specifically, comorbid psychosocial symptoms associated with alcohol misuse and CP make accurate identification of the etiology and treatment of distressing symptoms very complex. For example, high levels of emotional distress reported by CP patients may be attributable to coping with a chronic painful medical condition (CP), a current or past history of alcohol misuse, or a combination of the two. Research has also documented a relationship between alcohol use and pain; ongoing use has the potential to exacerbatea CP patient's pain experience [20]. However, very little research exists that examines patients with nonalcohol-related CP and associated psychosocial distress. Additional research that explores the presentation and association of psychosocial variables unique to nonalcoholic-related CP is needed. 

A better understanding of the relationship between depressive symptoms, pain, and quality of life in patients with nonalcohol-related CP holds promise for improving pain management and the quality of life of this unique patient population. The present study explores the frequency with which participants with nonalcohol-related CP endorse depressive symptoms and examines the relationship between depressive symptoms, pain experience, and quality of life among participants. Consistent with previous research on the relationships between depression, pain, and quality of life among patients with other chronic medical conditions, it was hypothesized that individuals with nonalcoholic intractable CP who met criteria for significant depressive symptomatology would report higher pain ratings and lower quality of life scores compared to those who did not meet criteria.

## 2. Methods

### 2.1. Ethics

All data were collected with full approval from the Institutional Review Board at the Medical University of South Carolina. Participants' personal health information was handled ethically and in accordance with Health and Human Services regulations. 

### 2.2. Subjects

The participants in this study consisted of 692 patients with nonalcohol-related, intractable pancreatitis. Participants had been diagnosed with pancreatitis for at least 6 months. The sample on average was middle age (*M *= 52.6, SD = 14.7), and 59% of the sample were female. 

### 2.3. Procedure

Data were collected as part of routine clinical care with patients who were being consecutively medically treated. Participants used a web-based computer psychosocial screening system to complete the measures at their initial visit to a tertiary care specialty clinic (Digestive Disease Center) at a large southeastern medical university. The participants completed the measures in a private physician office while they waited for their consultation with their physician. Nursing staff was available to help participants log-in to the system and to assist with completion of the online questionnaires if necessary.

### 2.4. Measures

 The following measures were completed by all participants.

(1) *The Medical Outcomes Trust Short Form 12 (SF-12)*. Health-related quality of life was assessed by administering the SF-12 [21]. The SF-12 is an abbreviated form of the SF-36 health status questionnaire [22], and it is a quality of life instrument that assesses mental (MCS) and physical (PCS) health and functioning over the past 4 weeks. The reliability and validity of the SF-12 have been well established [21], and the instrument has been validated for use in patients with chronic pain [19, 23–25]. The physical and mental quality of life scales are computed using the 12-items and range from 0 to 100 with a score of 0 indicating the lowest quality of life and a score of 100 indicating the highest quality of life.
(2) *The Center for Epidemiologic Studies 10-Item Depression Scale (CESD-10)*. The CESD-10 was used to assess self-reported depressive symptoms. This is a 10-item self-report measure with a 4-point rating scale (0–3) and has been demonstrated to be as effective as the full version of the CESD in identifying significant depressive symptoms [26–28]. Scores on the CESD-10 range from 0–30, with a score of 10 or greater indicating the presence of depressive symptoms.
(3) *The Brief Pain Inventory (BPI)*. The Brief Pain Inventory Short Form (BPI) is a 17-item self-report, multidimensional pain questionnaire that provides information about pain history, location, and intensity [29]. Participants are asked to rate the intensity of their pain experience on a scale of 0 (*no pain*) to 10 (*pain as bad as you can imagine*) at several time points: at its worst and its least over the past 24 hours, on average, and at the time of the assessment. For the purpose of the present study, participants only rated their pain on average. Although the BPI was first designed to evaluate cancer pain, it has since been validated in a chronic noncancer pain patient population [30].

### 2.5. Statistical Analyses

All study analyses were conducted with SPSS, version 12. Statistical significance was set at *P *= 0.05. Descriptive analyses were conducted, and Pearson correlations were run among the group as a whole examining the association between depression, quality of life, and pain on average. In addition, to further explore the relationship among variables, participants were divided into two groups: those that met criteria for the presence of depressive symptoms (score of 10 or higher on CESD 10) and those who did not meet criteria for presence of depressive symptoms. Independent sample *t-*tests were used to explore the differences between groups in quality of life (SF-12) and their rating of pain on average. If participants skipped any items they were excluded pairwise from each relevant analysis.

## 3. Results

The mean age of all participants was 52.6 (SD = 14.7); 41% of the sample were men. Demographic variables were not significantly related to depressive symptoms, pain, or quality of life. Correlation data for variables analyzed are summarized in Table 1. As predicted, participants' reports of depressive symptoms were significantly and positively related to ratings of pain on average (*r* = 0 .46, *P* < 0 .0001).

Similarly, participants' reports of depressive symptoms were significantly and inversely related to ratings of physical quality of life (*r* = −.22, *P* < 0.0001) and mental quality of life (*r* = −0.37, *P* < 0.0001). 

The mean CESD-10 score of the sample was 10.6 (SD = 6.5) with 52% of participants scoring above the cutoff for clinical depressive symptomatology. The average CESD-10 score for participants who scored above the clinical cutoff for depressive symptoms was 15.76 (SD = 4.34) compared to a mean of 4.96 (SD = 2.68) among the group scoring below the cutoff for depressive symptoms. Participants who scored above the clinical depression cutoff endorsed significantly more pain on average compared to those who did not meet the criteria for depressive symptoms. Specifically, participants above the depressive symptomatology cutoff rated their pain on average as 5.5 (out of 10; SD = 2.5), whereas those below the cutoff rated their pain on average as 3.4 (SD = 2.9; *t*(691) = 9.9, *P* < 0.0001). Moreover, participants above the cutoff on the CESD-10 rated their quality of life as significantly lower compared to participants below the cutoff, The mean physical quality of life norm-based *t*-score for those above the depressive symptomatology cutoff was 34.2 (SD = 7.6) on the physical quality of life subscale of the SF-12, and for those below the cutoff the mean physical quality of life was 37.6 (SD = 8.2; *t*(634) = 5.3, *P* < 0.0001). Similarly, the mean mental quality of life norm-based *t*-score for participants who scored above the cutoff was 44.9 (SD = 8.2), and for those below the cutoff, it was 49.0 (SD = 7.3; *t*(634) = 5.3, *P* < 0.0001). Results are summarized in Table 2.

## 4. Discussion

Chronic pancreatitis is a debilitating disease characterized by severe pain that negatively affects quality of life. While the etiology of the pain associated with chronic pancreatitis is not well-understood, alcoholism is the presumed cause in 55–80% of patients [31]. Assessing, treating, and managing patient's with alcohol-associated CP is difficult as a result of the myriad of psychosocial problems related to alcohol dependency, such as depression. A unique contribution of the current study is the large sample size comprised of patients with nonalcohol-related intractable CP, as this removes the confounding variable of alcohol dependency. 

 Results from the current study suggest that individuals who endorse more depressive symptoms also report more pain on average and lower physical and psychological quality of life. Further, findings from the present study indicate that many patients with nonalcoholic chronic pancreatitis (52%) experience depressive symptoms. Importantly, participants who endorsed significant depressive symptoms tended to also report worse pain and lower physical and psychological quality of life compared to those who did not acknowledge significant depressive symptoms. 

Findings lend support for the importance of assessing and treating CP patients using a biopsychosocial model. This treatment approach posits that biological, psychological, and social factors all significantly impact individuals' level of functioning within the context of chronic illness. This model has received increasing attention and support in the literature for understanding the etiology, course, and treatment planning for medical illness [31]. 

Current findings highlight the importance of the treatment of depressive symptoms in improving pain experience and physical and mental quality of life. A biopsychosocial model of treatment that targets symptoms of depression may include use of antidepressants and cognitive-behavioral interventions of activity-pacing, cognitive restructuring, and relaxation strategies. Environmental interventions may focus on increased communication, utilization of social resources, and physical rehabilitation. This comprehensive approach to treatment addresses the physiological, psychiatric, and social variables that are associated with increased distress in patients with CP.

The present study also highlights the potential value of early intervention and ongoing assessment of psychosocial variables in reducing depressive symptoms and improving pain and the general well-being of CP patients who are not alcohol dependent. The use of clinical interviews and self-report measures can provide valuable information to the treatment team related to the unique challenges that individual CP patients face and treatment can be tailored accordingly. Importantly, initial and ongoing psychosocial assessment provides physicians with information related to patients' needs for referrals to other specialists, such as mental health providers. 

There are several limitations to the present study. The current analysis was of retrospective data, limiting variables to the confines of prior data collection. Further, as this is a cross-sectional study, findings identify an association between depression, pain level, and quality of life; the direction of this relationship is unknown. Future prospective research that includes a theoretical design, experimental methods including healthy controls and participants with alcohol-related pancreatitis, and longitudinal designs are likely to further delineate the role of depression in the course of CP. The current study is also somewhat limited by the lack of examination of other psychosocial variables. It is possible that variables such as level of social support, coping style, substance use, and treatment history also play a role in patients' report of depressive symptoms, pain experience, and quality of life. Additional research that explores the possible mitigating role of these variables will provide valuable information related to the complexity of the relationship between depression, pain, and quality of life in CP patients. Further, more comprehensive measurement of the frequency, intensity, and quality of pain will offer a more nuanced picture of CP patients' experience. 

 In conclusion, findings suggest that among patients with nonalcohol-related CP depression is common and may be a risk factor associated with increased pain and decreased quality of life. Thus, routine screening and intervention for depression among patients with nonalcohol-related chronic pancreatitis may be warranted.

## Figures and Tables

**Table 1:** Correlations among variables tested in hypotheses.

	1	2	3
(1) Center for Epidemiological Studies Depression Scale-10	—		
(2) Pain on average	0.46 **	—	
(3) Physical quality of life (SF-12)	−0.22 **	−0.17 **	—
(4) Mental quality of life (BPI)	−0.38 **	−0.18 **	−0.40 **

^∗∗^ *P* < 0.0001.

**Table 2:** Independent *t-*tests between participants who met cut-off criteria for depressive symptoms and those who did not meet symptoms criteria and quality of life and pain on average.

	Depressive symptoms	No depressive symptoms	*t*	*P*
Physical QOL^a^	34.2 ± 7.6	37.6 ± 8.2	5.3	0.0001
Mental QOL^b^	44.9 ± 8.2	49.0 ± 7.3	5.3	0.0001
Pain^c^	5.5 ± 2.5	3.4 ± 2.9	9.9	0.0001

*N* = 691. However, the sample size for some of the variables is smaller due to missing data. ^a^ *n* = 610. ^b^ *n* = 610. ^c^ *n* = 666.
